# Cardiovascular reactivity, stress, and physical activity

**DOI:** 10.3389/fphys.2013.00314

**Published:** 2013-11-07

**Authors:** Chun-Jung Huang, Heather E. Webb, Michael C. Zourdos, Edmund O. Acevedo

**Affiliations:** ^1^Department of Exercise Science and Health Promotion, Florida Atlantic University, Boca Raton, FL, USA; ^2^Department of Kinesiology, Mississippi State University, Starkville, MS, USA; ^3^Department of Health and Human Performance, Virginia Commonwealth University, Richmond, VA, USA

**Keywords:** psychological stress, obesity, physical activity, microvascular reactivity, inflammation, resistance exercise, oxidative stress, stress hormones

## Abstract

Psychological stress has been proposed as a major contributor to the progression of cardiovascular disease (CVD). Acute mental stress can activate the sympathetic-adrenal-medullary (SAM) axis, eliciting the release of catecholamines (NE and EPI) resulting in the elevation of heart rate (HR) and blood pressure (BP). Combined stress (psychological and physical) can exacerbate these cardiovascular responses, which may partially contribute to the elevated risk of CVD and increased proportionate mortality risks experienced by some occupations (e.g., firefighting and law enforcement). Studies have supported the benefits of physical activity on physiological and psychological health, including the cardiovascular response to acute stress. Aerobically trained individuals exhibit lower sympathetic nervous system (e.g., HR) reactivity and enhanced cardiovascular efficiency (e.g., lower vascular reactivity and decreased recovery time) in response to physical and/or psychological stress. In addition, resistance training has been demonstrated to attenuate cardiovascular responses and improve mental health. This review will examine stress-induced cardiovascular reactivity and plausible explanations for how exercise training and physical fitness (aerobic and resistance exercise) can attenuate cardiovascular responses to stress. This enhanced functionality may facilitate a reduction in the incidence of stroke and myocardial infarction. Finally, this review will also address the interaction of obesity and physical activity on cardiovascular reactivity and CVD.

## Introduction

Chronic psychological stress is a risk factor for cardiovascular disease (CVD). In addition, acute psychological stress is asssociated with factors that explain the development of atherosclerosis; endothelial dysfunction, inflammatory reactivity and oxidative stress. The American Psychological Association has provided evidence that 20% of Americans report extreme stress and 80% report that their stress levels have increased over the past year (APA, 2012). Additionally, over the past 5 years, 60% of Americans have attempted to reduce their stress, with just 7% reporting success in reducing stress (APA, 2012). Examinations of acute responses to psychological stress provide insight into the potential mechanisms that may explain the relationship of psychological stress to CVD. Greater understanding can also provide support for considering therapeutic alternatives that may alleviate the ill effects of stress.

**Figure 1:**
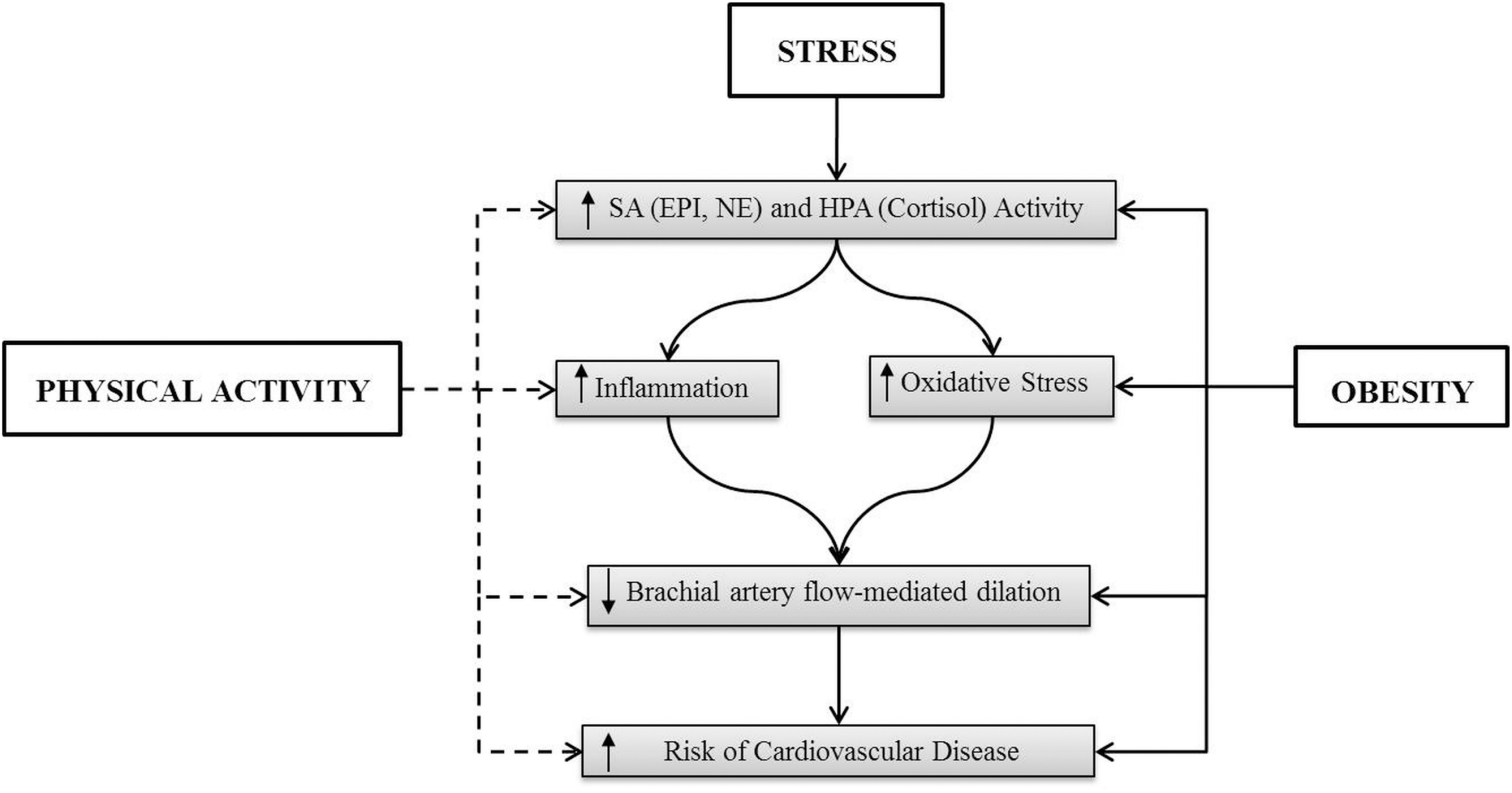
**The solid line indicates an activation of the indicated factor and the dashed line represents the suppression of the indicated factors**. The effect of stress in obesity has not been delineated.

This review will examine stress-induced cardiovascular reactivity and plausible explanations for how exercise training and physical fitness (aerobic and resistance exercise) can attenuate cardiovascular responses to stress. Important to our understanding of the development of CVD is how the benefits of physical activity in attenuating the cardiovascular stress response (enhanced functionality) may also support a reduction in the incidence of stroke and myocardial infarction. Finally, in light of the high prevalence of overweight and obesity (68.8% of US adults were categorized as overweight in 2008, and 35.7% were categorized as obese (Flegal et al., 2012) and support for the concept that obesity can be considered a chronic stressor marked by chronic inflammation, oxidative stress, and endothelial dysfunction, this review will also address the interaction of obesity and physical activity on cardiovascular reactivity and CVD (Figure 1).

## Sympathoadrenal responses to acute stress and adaptation to physical activity

Initially, the neuroendocrine response to stress was believed to be attributable solely to the release of catecholamines from the adrenal medulla (Cannon and De La Paz, 1911; Cannon, 1914). Cannon and De La Paz (1911) research regarding sympathetic activation in response to threat or danger resulted in the introduction of the concept known as the “fight or flight” response. Furthering Cannon's work, Hans Selye (1936) conceptualized that factors such as heat or cold, forced immobilization or exercise, as well as chemical, biological, and psychological factors will elicit the exact same non-specific response of not only the catecholamines, but also corticosteroids (Cannon and De La Paz, 1911; Cannon, 1914; Selye, 1936, 1950, 1954, 1976).

The relationship between psychological stress and cardiovascular reactivity has long been suggested as an explanation for the association between psychological stress and CVD. It is known that chronic psychological stressors can lead to increased risk of arteriosclerosis, hypertension, and other metabolic disorders (Chrousos, 2000b, 2009; McCrone et al., 2001; Kyrou and Tsigos, 2009), while acute stressors result in acute increases in blood pressure, HR, and decreased metabolic efficiency (Crews and Landers, 1987; Hamer et al., 2006; Webb et al., 2011).

When an individual is psychologically or physiologically stressed in an acute manner, a complex chain of reactions occur, stemming from responses occurring within the sympathoadrenal (SA) and hypothalamic pituitary adrenal (HPA) axes, and the parasympathetic (PNS) and sympathetic nervous system (SNS) pathways in the body. Acute psychological stress has been shown to elicit increases in the secretion of epinephrine (EPI) and norepinephrine (NE) from the SA axis (Frankenhaeuser, 1991; Schoder et al., 2000; Gerra et al., 2001), and cortisol from the HPA axis (Frankenhaeuser, 1991; Chrousos, 2000a; Gerra et al., 2001). It has also been suggested that the increases in HR and BP response are due to a decrease in vagal tone attributed to the PNS as a result of rhythmic shifts in HR mediated by the brainstem medullary mechanisms through the vagus nerve (Hatfield et al., 1998; Spalding et al., 2000; Smeets, 2010) or an increase in afferent sympathetic neuromuscular activation (Kaufman and Hayes, 2002; Smith et al., 2006). Physical stressors, such as exercise, will elicit NE release in a curvilinear manner in response to increasing workloads, while EPI secretion increases at workloads above 60% of an individual's VO_2max_ (Hjemdahl, 1993; Rowell and Shepherd, 1996), and these increases are likely responsible for the concomitant increases in HR and BP.

While these same physiological mechanisms are experienced in response to a stressor of any type, the amount of reactivity and type of response experienced has been suggested to be impacted by multiple factors, including an individual's perception of control over a situation, the combination of multiple stressors, the level of cardiorespiratory fitness, obesity levels, and sex. The greater the amount of control an individual perceives they have over a situation results in lesser catecholamine, HR, and BP responses relative to a situation where an individual feel less control over a situation (Frankenhaeuser et al., 1976; Frankenhaeuser, 1991; Hinton et al., 1991).

While the majority of investigations of stress are often addressed from a unidirectional perspective, it has been suggested that psychological appraisals can interact with and cause alterations in peripheral physiological responses. The proposed transactional psychobiological model of cognitive appraisal during exercise suggests an individual's perception of the demands imposed by psychological and physical stressors is related to an individual's perceived ability to meet these demands Acevedo and Ekkekakis (2001). The combination of mental and physical stress has been shown to result in an exacerbated SA (Roth et al., 1990; Szabo et al., 1994; Rousselle et al., 1995; Acevedo et al., 2006; Huang et al., 2010a; Webb et al., 2010, 2008) and HPA (Webb et al., 2008, 2011, 2013) responses above that of a single stressor alone. Therefore, if pathological events are related to psychological and physical stress independently, then this combination of stressors, resulting in an exacerbated SA and HPA response, are likely be responsible for greater pathophysiological alterations in these systems.

Exercise is also believed to have an attenuating effect on an individual's reactivity level at resting levels, and research has shown this to generally be true (de Geus and van Doornen, 1993; de Geus et al., 1993; Porges, 1995; Sothmann et al., 1996; Schuler and O'Brien, 1997), with individuals of higher fitness levels exhibiting a lesser HR response to psychological stress (Claytor, 1991; Boutcher and Nugent, 1993; Spalding et al., 2000), as well as an attenuated EPI and NE response (Boutcher and Nugent, 1993). It has also been shown that PNS responses increase after exercise and this response may assist in blunting responses that elicit increases in HR and BP (Porges, 1995; Dishman et al., 2002). Exercise may have be beneficial effects on HPA functioning because of lower stress-induced cortisol responses in physically fit compared with unfit subjects (Traustadóttir et al., 2005; Rimmele et al., 2009). However, the actual impact of cardiorespiratory fitness on cardiovascular reactivity is still a topic of debate as multiple meta-analyses and larger clinical studies investigating this topic have differed in their conclusions (Crews and Landers, 1987; Forcier et al., 2006; Hamer et al., 2006; Jackson and Dishman, 2006; Sloan et al., 2011; Alex et al., 2013), with the studies citing the differences in participant demographics, research methodologies, and measured variables adding to the difficulty in coming to definitive conclusion.

Similarly, studies investigating the relationship between adiposity and SA axis responses also lack a clear relationship, with inconsistent relationships found among obesity and catecholamine response (Macdonald, 1995), although there is support for an increase in SNS reactivity in obesity (Grassi et al., 1995; van Baak, 2001; Alvarez et al., 2002). Abdominal obesity has been associated with both exaggerated (Goldbacher et al., 2005; Steptoe and Wardle, 2005) and blunted (Hamer et al., 2007; Carroll et al., 2008; Phillips, 2011; Phillips et al., 2012) cardiovascular reactivity. Thus, these differences in findings may be related to a number of factors, including participant demographic differences and methodological issues. It can be suggested that generally, studies with appropriate statistical adjustments and stringent inclusion criteria have reported negative associations between cardiovascular reactivity and abdominal obesity (Laederach-Hofmann et al., 2000; Hamer et al., 2007; Phillips, 2011).

It is also interesting to note that while both males and females react to stress through the same psychophysiological pathways, they do so with markedly different results. Research demonstrates that males respond with greater diastolic blood pressure and total peripheral resistance changes during acute stressors (Stoney et al., 1987, 1988; Matthews and Stoney, 1988; McAdoo et al., 1990; Lai and Linden, 1992; Matthews et al., 1992; Allen et al., 1993), while females experience greater changes in HR during a psychologically stressful situation (Frankenhaeuser et al., 1976; McAdoo et al., 1990; Frankenhaeuser, 1991; Allen et al., 1993). In addition to cardiorespiratory differences, it has also been shown that when subjected to an acute psychological stress, males had a significantly greater rise in EPI levels in comparison to females', whose EPI rose slightly or not at all. A similar, but less pronounced sex difference was also found for NE, with males again registering a greater change in hormonal levels (Frankenhaeuser et al., 1978; Forsman and Lindblad, 1983; Lundberg, 1983). These differences have led to suggestions (Allen et al., 1993) that male and female responses to mental stress may be attributed to different mechanisms, with males being considered “vascular” reactors and females classified as “cardiac” reactors.

One mechanism that has been suggested to account for differences in male and female vascular response during psychological stress is a greater sensitivity in peripheral alpha- and beta-adrenergic receptors in women (Freedman et al., 1987; Girdler et al., 1990; Kajantie and Phillips, 2006). Another proposed mechanism that may account for the larger cardiac response in women may be a greater sensitivity and/or density of adrenergic receptors in the myocardium (Girdler et al., 1990; Kajantie and Phillips, 2006). Both hypotheses have been supported by research showing women respond with lower catecholamine secretions during an acute psychological stress when compared to males (Frankenhaeuser et al., 1978; Rauste-von Wright et al., 1981; Collins et al., 1985). It was also noted that differences in cortisol secretions during psychologically stressful tasks are negligible between males and females (Frankenhaeuser et al., 1978; Forsman and Lindblad, 1983; Lundberg, 1983), further suggesting that the SA axis may play a key role in reactivity differences among males and females during an acute psychological stress.

In addition, it should be noted that a number of studies have shown that men have higher BP levels than women through much of their lifetime regardless of race and ethnicity (Sandberg and Ji, 2012). Particularly, acute stress results in immediate increases in arterial blood pressure (Lutgendorf et al., 2000) to be a result of vasoconstriction prompted by enhanced SA activity (McCarty and Gold, 1996). Furthermore, chronic stress may lead to hypertension and other cardiovascular dysfunction as a result of disturbances in the SA axis and the nitric-oxide pathways (Alvarez et al., 2001; Stefano et al., 2001; Esch et al., 2002).

Interestingly, several studies demonstrate that cardiovascular responses during mental stress are better predictors of future hypertension (Wood et al., 1984; Matthews et al., 1993) than resting BP measurements. Furthermore, because an individual's BP levels respond to many factors, including daily activities and affect (Pickering, 1997), it has been hypothesized that cardiovascular responses during mental challenges may be better predictors of future resting BP levels than BP at rest. Further, it has been reported that BP levels during mental stress are more closely associated with left ventricular mass than are resting BP levels (Georgiades et al., 1996).

These acute responses to mental challenge can elicit increased cardiac output, increased systemic vascular resistance, and thus an elevation of arterial blood pressure. While sympathetic elevations that are transient in nature prepare the body for accommodating to possible physical demands that an individual may encounter, prolonged or frequently occurring elevations of the catecholamines can result in vasoconstriction in most systemic arteries and veins leading to allostatic alterations in cardiovascular responses (Gidron et al., 2002). These alterations provide conditions favorable for the development of hypertension, endothelial dysfunction, and may contribute to the development of arteriosclerosis (Chrousos and Gold, 1998; Gidron et al., 2002; Spieker et al., 2002).

Research has shown psychological stress may be attenuated by a number of factors including exercise (Crews and Landers, 1987; van Doornen and de Geus, 1989; de Geus et al., 1993; Sothmann et al., 1996; Schuler and O'Brien, 1997; Acevedo et al., 1999). Additionally, exercise has been shown to have immediate psychological benefits comparative to those of other traditional therapeutic modalities and is a more effective anxiolytic agent than cognitive therapies when assessing anxiety (Petruzzello et al., 1991). This proposed explanation is also supported by Dienstbier's (1989) physiological toughness hypothesis, in that it is suggested that cognitively based stress reduction strategies only provide a short-term solution to anxiety reduction.

## Microvascular reactivity to oxidative stress and inflammation

Chronic stress has been demonstrated to be a determinant of CVD (Olinski et al., 2002). One of the earliest sub-clinical stages in the atherosclerotic process is an impairment of endothelium-dependent vasodilation, also known as endothelial dysfunction (Singhai, 2005). Acute mental stress is capable of altering physiological homeostasis such as microvascular reactivity (Huang et al., 2013; Szijgyarto et al., 2013). Laboratory-induced psychological stress has been demonstrated to induce transient endothelial dysfunction [impairment of brachial artery flow-mediated dilation (FMD)] (Ghiadoni et al., 2000; Szijgyarto et al., 2013). This stress-induced impaired FMD has been shown to be worsened in high-stress occupations (e.g., firefighting, law enforcement) and patients with stress disorders (e.g., depression) (Violanti et al., 2006; Mausbach et al., 2012; Wagner et al., 2012). Fahs et al. (2009) found increased aortic and carotid artery stiffness in firefighters. This observation has also been discovered following 3 h of firefighting activities (Fahs et al., 2011a,b). Furthermore, Joseph et al. (2010) showed a lower FMD in police officers compared to controls. This impaired FMD in police officers was associated with decreased carotid intima-media thickness, and 16 and 36% of these police officers (*N* = 100) met criteria for depression and reported posttraumatic stress disorder symptoms, respectively (Violanti et al., 2006).

A number of mechanisms have been shown to be involved in endothelial dysfunction that occurs as a result of acute mental stress. For example, Broadley et al. (2005) showed that acute mental stress-induced endothelial dysfunction (lower FMD) was prevented with an oral administration of metyrapone (an inhibitor of cortisol synthesis) in healthy individuals. Furthermore, plasma cortisol levels are higher at rest and in response to acute mental stress in firefighters and police officers compared to controls (Tomei et al., 2003; Rosati et al., 2011; Robinson et al., 2013). These reported elevations in cortisol levels have been associated with impaired FMD in police offices (Violanti et al., 2009). These findings suggest that the challenges of the HPA axis experienced by high-stress occupations may lead to an increased risk of CVD. Although the underlying mechanisms remain to be determined, elevated levels of oxidative stress and inflammation have also been implicated as contributing factors that link acute mental stress to endothelial dysfunction.

A mediator of endothelial dysfunction is shear stress (a dragging frictional force generated by blood flow in the vasculature), leading to oxidative stress (Bagi et al., 2003). Oxidative stress is an imbalance between antioxidants [e.g., nitric oxide (NO)] and reactive oxygen species (ROS) [superoxide (O2^−^), hydrogen peroxide (H_2_O_2_), and hydroxyl radical (OH^−^)] (Sies, 1997). In healthy vascular cells, ROS is generated during the metabolism of oxygen, with the rate of ROS production being balanced by the rate of oxygen elimination (Vider et al., 2001). When ROS production is elevated, the process of cell damage occurs and can possibly facilitate the development of CVD (Ji et al., 2006). Furthermore, any increase in vascular shear stress from the cardiovascular alterations in response to a mental or physical perturbation can influence the balance of oxidative stress.

Research has previously shown that psychological stress may contribute to the development of atherosclerosis by eliciting an elevation in ROS, which can further induce oxidative DNA damage (Olinski et al., 2002). Subsequently, in a study on medical students, Sivonova et al. (2004) demonstrated greater nuclear DNA damage in lymphocytes on the day of an examination (stress condition) compared with during the time between two examination periods (non-stress condition). To further understand endothelial dysfunction by oxidative stress, Chung et al. (2010) recently examined the effects of immobilization stress (120 min/day for 14 days) in rats and found that arterial endothelial nitric oxide synthase (eNOS) mRNA and NO decreased and plasma malondialdehyde level (a marker of oxidative stress) increased along with decreased acetylcholine-induced relaxation of arteries as compared to controls. Interestingly, Huang et al. (2010a) examined oxidative stress response in healthy individuals who were exposed to acute dual challenge (physical and psychological stress). This study found that the dual challenge condition elicited greater plasma 8-isoprostane levels (a biomarker of oxidative stress) compared to exercise alone condition. This finding may provide the potential explanation that a combined physical and psychological stress often experienced chronically by occupational professionals (e.g., emergency responders, firefighters, and police officers) is a contributing factor to endothelial dysfunction, thereby increasing the risk of CVD.

In addition, vascular inflammation plays a critical role in endothelial dysfunction which can be induced by the production of pro-inflammatory cytokines such as tumor necrosis factor-alpha (TNF-α) and interleukin-6 (IL-6) (Ross, 1999; Esteve et al., 2007). Notably, a previous study has suggested that individuals who have a greater laboratory-induced stress response are more likely to experience higher stress in daily life (Wirtz et al., 2008), and this response is associated with increased risk for atherosclerosis (Kamarck et al., 1997). An increase in circulating TNF-a and IL-6 has been observed following acute mental stress (Steptoe et al., 2001; Heinz et al., 2003) via activation of nuclear factor-kappa B (Barnes and Karin, 1997; Bierhaus et al., 2003). Furthermore, Huang et al. (2010b) found a greater plasma IL-2 levels in firefighters who experienced a dual stress (physical and psychological stress) compared to exercise alone group. It is important to note that the magnitude and direction of inflammatory cytokine response to psychological stress vary and are dependent on the acute or chronic nature of the stimuli. For example, Kang and Fox (2001) examined chronic academic stress during examinations and found that decreased IL-2 [in both peripheral blood mononuclear cell (PBMC) and whole blood measures] and IFN-g (only PBMC) levels were observed, whereas an elevation was seen in IL-6 (in both PBMC and whole blood measures). In a recent study, Ramey et al. (2012) examined the inflammatory cytokine response in law enforcement officers and found that the job demand (physical and psychological) assessed by the Job Content Questionnaire was positively associated with resting IL-1 beta and IL-6. This finding is further supported by Groer et al. (2010) who demonstrated a significant increase in salivary IL-6 in police officers following simulated workplace scenario (6 min of tracking a gunman through a building. It is important to note that ~23% of law enforcement officers who reported metabolic syndrome were physically inactive (Yoo et al., 2009). In a cross-sectional study of 527 firefighters, Durand et al. (2011) demonstrated the CVD risk factors such as triglyceride and low density lipoprotein cholesterol levels are negatively associated with time and frequency of physical activity per week. These elevations in the mediators of endothelial dysfunction may be partially explained by physical inactivity. Thus, it is pivotal to understand how lifestyle changes such as exercise may help alleviate the potential for the negative health outcomes in these occupations.

Epidemiological evidence consistently shows that the benefits of physical activity and fitness on physiological and psychological health. One of the purported benefits associated with aerobic fitness is the attenuation of the cardiovascular response during psychological stress and recovery (Dienstbier, 1989; Sothmann et al., 1996; Spalding et al., 2004). For example, aerobically trained individuals have shown to attenuate reactivity and recovery of HR and blood pressure (Crews and Landers, 1987; McCubbin et al., 1992) and lower cortisol reactivity to acute mental stress (Webb et al., 2013). Furthermore, aerobic exercise training has been shown to defend against ROS-induced lipid peroxidation and to decrease the occurrence of ROS-associated diseases such as CVDs and Alzheimer disease (Mattson and Wan, 2005; Perry et al., 2005; Radak et al., 2005). A number of studies have shown that exercise training can enhance the adaptation of oxidative stress by increasing antioxidant defenses demonstrated by the up-regulation of antioxidant gene expressions such as superoxide dismutase and glutathione peroxidase (Powers and Lennon, 1999; Leeuwenburgh and Heinecke, 2001). Additionally, Nojima et al. (2008) found that an oxidative stress marker (urinary 8-OHdG level) decreased in patient with type 2 diabetes following a 12-month of aerobic exercise training. These findings suggest that regular exercise is beneficial in up-regulating the resistance against oxidative stress.

In addition, physical fitness has been shown to provide a more resilient immune defense and greater stress protection. For example, a reduction in pro-inflammatory levels (TNF-a and IL-6) was found in patients with coronary heart disease following aerobic exercise training (Goldhammer et al., 2005), and lower IL-6 concentrations have been observed in individuals who had self-reported higher physical activity levels (Pischon et al., 2003). In response to acute mental stress, physical fitness is associated with diminished pro-inflammatory cytokine responses (TNF-α and IL-6) (Hamer and Steptoe, 2007). While stress management alone has not demonstrated an improvement in FMD (Blumenthal et al., 2005), Dod et al. (2010) have shown an improvement of FMD along with decreased IL-6 levels in patients with coronary artery disease (CAD) following 12-weeks of lifestyle interventions including exercise and stress management. Therefore, the development of strategies, including exercise training, to address the negative consequences of chronic stress may help alleviate the elevated risk for CVD.

## Resistance exercise: cardiovascular reactivity to stress

Resistance exercise has long demonstrated positive adaptations in relation to skeletal muscle hypertrophy, muscular strength, and body composition (Wilmore, 1974). However, the effects of resistance training on cardiovascular reactivity and acute and long-term markers of stress are significant and perhaps overlooked. To appropriately understand these adaptations, in-depth insight must be pursued to examine the psychological effects of resistance exercise, and varying hormonal (cortisol, EPI, and NE) and hemodynamic responses (HR and BP). Additionally, knowledge related to the blood flow and inflammatory cytokine response, which may occur as a result of structured and periodized resistance training programs designed to elicit a desired training adaptation.

Pioneer research from Morgan (1969) has demonstrated a greater incidence of depression in unfit individuals compared to those who are more physically fit. Since then a plethora of data has illustrated the benefits of exercise to be associated with lower emotional distress (Steptoe and Butler, 1996) and decreased levels of depression (Steptoe et al., 1997). Additionally, longitudinal data from Paffenbarger et al. (1994) found physical activity to be inversely correlated with depression over the course of 25 years in a sample of over 10,000 men. The confounding variable, however, is that the research related to mental health has mostly examined aerobic exercise activity and is limited regarding resistance exercise. However, chronic resistance training has been shown to cause reductions in resting concentrations of the stress hormone cortisol (Hakkinen et al., 1988; Kraemer et al., 1998). This factor is of great importance as increased resting cortisol levels have been associated with impaired declarative memory in healthy adults (Kirschbaum et al., 1996). Additionally, resistance training may be an attractive strategy over the life span to not only attenuate the onset of sarcopenia (the loss of type II fibers with age), but to prevent elevations in cortisol, which have been related to memory impairments in elderly humans (Lupien et al., 1996) and to predict hippocampal dysfunction in an aging population (Lupien et al., 1998). Therefore, even though the prominent research related to exercise and mental stress focuses on endurance training, a physiological analysis of the benefits associated with resistance training suggests positive outcomes for mental stress when resistance training is included in an individual exercise routine.

Commonly, analyzed hormonal response in regards to resistance training protocols includes the anabolic hormone testosterone and the catabolic hormone cortisol, which are secreted from the HPA axis. Via the endocrine system, secretion of these hormones in response to training occurs to maintain homeostasis in the body (Galbo, 1983). Fluctuations in these hormones can occur acutely in response to exercise, or resting concentrations may be altered as an adaptation to a chronic training stress (Fry et al., 1994; Deschenes et al., 1998). As an acute response, it is typical to for significant increases in testosterone and cortisol to occur, however, smaller changes in resting levels over the long-term may indicate a positive response to stress (Staron et al., 1994). Moreover, the literature has demonstrated that acute hormonal fluctuations are simply due to the metabolic stress response of physical activity and bear little to no impact on long-term muscle performance adaptations (Ahtiainen et al., 2005). Further, these acute changes may even lead to decreased stress and increased exercise performance over time (Hakkinen et al., 1988). Recently, investigators have demonstrated that an intense bout of resistance training (5 sets of 8 repetitions @75% of one repetitions maximum -1RM) significantly increases both the catabolic hormone cortisol and the anabolic hormone testosterone by a similar percentage (McCaulley et al., 2009). Additionally, the acute stress response seems to be augmented by total work performed or training volume (Sets X Repetitions X Weight Lifted). In response to long-term training, data has shown decreased cortisol levels to lead to greater levels of muscle performance (Staron et al., 1994; Kraemer et al., 1999). Furthermore, data exists demonstrating that long-term (2 years) resistance training may increase the testosterone to cortisol ratio (T/C ratio), which also results in an increase in muscular force development (Hakkinen et al., 1988). Therefore, a reduction in cortisol over time may be an important signal of resistance training adaptation and a decline in stress levels leading to increased exercise performance.

Another stress response to consider is the elevation and recovery paradigm of the neurohormones of the adrenal medulla: EPI and NE. A plethora of research has demonstrated that, in response to training, there are acute elevations in levels of EPI and NE similar to cortisol, signaling a stress response (Kraemer et al., 1987). This acute response shows elicited activation of the adrenal medulla, evidenced not only by increases of EPI and NE up to 15 min post-resistance training, but also a decrease in plasma peptide f (P-F) immediately following training (Bush et al., 1999). Data from Bush et al. (1999) also revealed that following the initial decrease of P-F, the hormone then reverses course and increases up to 4 h following a resistance exercise protocol of either 10 or 15 repetitions per set. This observation of elevated P-F for up to 240 min following exercise is likely demonstrating that the adrenal medulla serves a physiological function to maintain appropriate homeostasis. The prolonged increase in P-F is intriguing as other research has shown elevations only up to 15 min post-exercise (Kraemer et al., 1985a,b, 1990), but a prolonged increase may suggest that secretion of P-F from the adrenal medulla may aid in recovery via immune response. The relationship between P-F and immune response is plausible, as data has demonstrated P-F to enhance antibodies as a result of increased T-Cell function (Hiddinga et al., 1994). Ultimately, T-Cells then serve as a mediator for B-Cell stimulation and response capability (Hiddinga et al., 1994). Therefore, the prolonged increase in P-F may signal as a response to combat stress by serving as a signaling mechanism for enhanced immune system activity. Further, these authors reported that a group who performed 10 repetitions per set had greater force output and blood lactate than those who performed 15 repetitions per set, however, there was no difference in the acute hormonal stress response to exercise. This lack of difference is likely because total work was equated.

Further analysis of adrenal medulla secretions in response to resistance exercise reveals that in previously trained lifters EPI and NE seem to increase while P-F seems to decrease (Bush et al., 1999). Additionally, in trained weightlifters, there is a significant increase in P-F up to at least 240 min following exercise (Bush et al., 1999). This demonstrates an important training adaptation acting to increase a trainee's ability to combat stress. In other words, a trained lifter may fight the high physiological demands of resistance training with an increase in P-F to signal immune response leading to recovery. This training adaptation is of importance, as it is well established that the eccentric phase of resistance training leads to significant skeletal muscle myofiber damage and fatigue as evidenced by elevated plasma creatine kinase (CK) levels (Nosaka et al., 2001; Chen and Hsieh, 2005) and soreness (Nosaka et al., 2001). Concomitantly, as myofiber damage occurs an influx of neutrophils and blood monocyte secretion of IL-1 Beta into the muscle, this may last for up to 5 days (Cannon et al., 1989; Fielding et al., 1993). Therefore, elevated P-F for up to 4 h may augment the recovery process to attenuate the muscle damage response. Ultimately, total work seems to be the key factor to elicit neurohomronal response, however, training status may increase secretions of hormones from the adrenal medulla leading to an improved immune response and faster recovery.

Additionally, resistance training has frequently been used as a component of cardiac rehabilitation programs (Pollock et al., 2000) and has shown resistance training status to effect hemodynamic responses (i.e., HR and BP). It is well known that acute increases in HR and BP are significant during and following an intense bout of resistance training (Darr et al., 1988). While parasympathetic activity is responsible for the initial rise in HR during resistance training due to vagal withdrawal, it is the elevation in activity of the SNS during an intense strength training session, which is responsible for increasing HR. Interestingly, individuals with previous training experience have seen HR return to baseline levels sooner following exercise than those of a less-advanced training status (Darr et al., 1988). This response suggests that chronic adaptations to resistance training act to handle increasing levels of stress and cause a more rapid return of HR to baseline levels following an intense bout of resistance training.

Moreover, resistance training has been shown to be a safe mode of exercise in patients with CAD or congestive heart failure CHF (Karlsdottir et al., 2002), this supports the use of chronic resistance training as a means to decrease the risk of stroke and myocardial infarction. In support of this notion, Karlsdottir et al. (2002) reported that subjects with CAD and CHF increased HR and BP to similar levels as cycling at 90% ventilatory threshold when performing one set of 10 repetition at 60–70% of one-repetition maximum on either the biceps curl, leg press, or shoulder press exercises. Additionally, these authors reported the stability of left ventricular function with resistance exercise in cardiac patients. Finally, CAD and CHF patients show a similar pattern of hemodynamic responses following resistance training to that of healthy individuals. Therefore, including resistance training in a rehabilitation program for cardiac patients may be a key component and appropriate stress reducer designed to improve quality of life and increase muscle mass.

Recently, blood-flow restriction training (BFR) at rest and in conjunction with resistance exercise has become an increasingly attractive method in which to examine cardiovascular reactivity. BFR training has been shown to increase muscle strength and hypertrophy by restricting blood flow proximal to the exercising muscle resulting in blood pooling and a restriction of venous return (Iida et al., 2007; Loenneke et al., 2012). It has been demonstrated that even without exercise, BFR training via application of a KAATSU device with a pressure of 200 mmHg has produced HR and BP responses similar to that of an orthostatic impetus (Iida et al., 2007). These intriguing results suggest that BFR may be a plausible method to be utilized as a countermeasure against orthostatic intolerance. Interestingly, recent data suggest that the introduction of BFR with resistance exercise has demonstrated greater HR and BP during exercise in young (30 years) and older (66 years) subjects (Vieira et al., 2013). However, in healthy individuals, BP and arterial compliance did not change following 6 weeks of full body resistance training in a low-intensity BFR resistance exercise group (20% of 1RM), nor did it change in moderate-intensity (70% of 1RM) or low-intensity (50% 1RM) resistance exercise groups (Fahs et al., 2011a,b). Therefore, individuals who have contraindications to performing high-intensity resistance training, such as cardiac patients, may be able to get resistance training benefits while not negatively altering arterial compliance by using blood flow restricted exercise.

In addition to the catabolic hormone cortisol discussed previously, the pro-inflammatory cytokine, IL-6, and anti-inflammatory cytokine, IL-10, can be important factors to alert of a stress response and subsequent fatigue and recovery. Because resistance exercise causes myofibrillar disruption and localized edema, an inflammatory response mediated by cytokines will occur (Izquierdo et al., 2009). It is therefore likely that the response of inflammatory cytokines in the presence of skeletal muscle damage serve as a mediator of satellite cell activation and myofiber repair to reduce stress and induce hypertrophic adaptations of the skeletal muscle (Izquierdo et al., 2009). Data has shown IL-6 to be elevated up to 48 h post-resistance training of 5 sets of 10 repetitions (Izquierdo et al., 2009). This elevation in IL-6 is consistent with research showing increased CK levels at 48 h following similar resistance protocols (Chen and Hsieh, 2005) and suggesting that IL-6 is directly correlated with the muscle damage response, further signifying the role of IL-6 in recovery and its importance in alleviating a stress response to training (Smith et al., 2000; Suzuki et al., 2002; Izquierdo et al., 2009).

The anti-inflammatory cytokine IL-10 has been increased by catecholamines (EPI and NE) following exercise (Peake et al., 2005). Further, the responsiveness of IL-10 seems to be augmented in a subsequent training bout 4 weeks later (Hirose et al., 2004). The greater response of IL-10 in a subsequent training bout may be viewed as an adaptation to stress as a result of decreased inflammation. The attenuated inflammatory response to training is a component of the repeated bout effect (RBE), which stipulates that when the same exercise (Nosaka et al., 2001) is performed in a subsequent resistance training bout, the muscle damage response is attenuated. Ultimately, IL-6 and IL-10 not only play important processes in recovery, but their blunted release secondary to chronic training can be a demonstration of the RBE and may signify a reduction in the stress response to resistance training.

Data has demonstrated the ability of resistance-trained individuals to improve adaptations to stress, including; lower cortisol levels (Hakkinen et al., 1988), a more rapid return of HR to baseline levels (Darr et al., 1988), and increases in P-F possibly leading to enhanced immune function (Bush et al., 1999). Moreover, a reduction in cortisol levels that is associated with chronic resistance training is likely an appropriate method to attenuate decreasing hippocampal function with aging. Additionally, resistance training seems to be a safe method for patients with CAF and CAD (Karlsdottir et al., 2002) and its benefits may decrease the risk of CVD. Ultimately, for an individual to reduce chronic stress it seems that resistance training is a key component to optimize this goal.

## Future research directions: endothelial function to stress in obesity

Obesity is associated with job-associated stress (tension and anxiety) and depression, and these stress related disorders have been found to lead to an increased risk of CVD and mortality (Nishitani and Sakakibara, 2006; Valtonen et al., 2012). Recent studies have reported ~77% overweight and obesity rates in high-stress professionals such as young emergency responders (firefighters and ambulance recruits), police officers, and military personnel (Franke et al., 2002; Hsu et al., 2007; Tsismenakis et al., 2009; Ramey et al., 2009). Importantly, obesity has been shown to disturb cardiovascular responsivity to acute mental stress (Hamer et al., 2007), which may associate with stress-related endothelial dysfunction. Furthermore, in response to acute mental stress, Ghiadoni et al. (2000) have also shown that diabetic patients have a lower FMD compared to the control subjects. In addition, Martin et al. (2013) found that the index of adiposity (e.g., BMI, waist circumference, and waist-to-hip ratio) were negatively correlated with measures of vascular function such as hyperemic velocity time integral and hyperemic shear stress in a total of 1462 male firefighters. Thus, understanding the link between obesity and psychological stress may provide a critical contribution in determining the pathopysiological mechanisms of CVD development.

Although there is limited information investigating the impact of psychological stress on the oxidative stress in obese populations, one mediator of obesity-induced endothelial dysfunction may be the level of oxidative stress (Timimi et al., 1998; Schäfer and Bauersachs, 2008). Flint et al. (2007) has demonstrated that cortisol, norepinephrine, and epinephrine released during psychological stress can induce DNA damage within 10 min. A study by Epel et al. (2000) showed that elevated cortisol responses to acute psychological stress are associated with increased waist-to-hip ratio. This increase in oxidative stress during psychological stress could possibly be the negative effect of high circulating levels of stress hormones (e.g., cortisol).

Another potential mechanism to explain stress-induced endothelial dysfunction in obesity is the interactions of leptin with oxidative stress and inflammation. Leptin, an adipocyte-derived hormone, plays an important role in metabolism, adiposity, and vascular inflammation, and has been implicated in the development of coronary heart disease (Wannamethee et al., 2007). *In vitro* stimulation of cultured human endothelial cells with leptin has induced an increased accumulation of ROS and levels of pro-inflammatory mediator (e.g., monocyte chemotactic protein-1) via activation of nuclear factor-kappa B (Bouloumie et al., 1999). Interestingly, recent research has shown that people who undergo acute psychological stress demonstrate increases in leptin levels, and these increases are positively correlated with waist circumference (Otsuka et al., 2006; Brydon et al., 2008). Brydon et al. (2008) also showed that a positive correlation between basal circulating leptin and IL-6 responses in response to mental stress. Taken together, these findings suggest that stress-induced leptin may partially contribute to the development of endothelial dysfunction. However, although a number of investigators have expressed interest in examining the mechanisms underlying psychological stress-induced endothelial dysfunction, the possible interaction (additive or synergistic) of obesity and psychological stress on the development of endothelial dysfunction are still not fully understood. Future investigation should attempt to expand the understanding of the mechanisms contributing endothelial dysfunction that links between obesity, psychological stress and CVD.

## Conclusions

The studies that have been conducted examining the effect of physical activity on cardiovascular reactivity and CVD generally support Dienstbier's physiological toughness hypothesis (1989). More specifically, physical activity can not only reduce the immediate effects of stress but can also enhance the recovery from stressors (Crews and Landers, 1987; van Doornen and de Geus, 1989; de Geus et al., 1993; Sothmann et al., 1996; Schuler and O'Brien, 1997; Acevedo et al., 1999). Additionally, exercise has been shown to have immediate psychological benefits relative to other therapeutic treatments and can likely serve as a very effective adjuvant therapy (Petruzzello et al., 1991).

Furthermore, aerobic exercise training has been shown to defend against ROS-induced lipid peroxidation and to decrease the occurrence of ROS-associated diseases such as CVDs and Alzheimer disease (Mattson and Wan, 2005; Perry et al., 2005; Radak et al., 2005). These findings suggest that regular exercise is beneficial in up-regulating the resistance against oxidative stress. The immune system has also demonstrated positive adaptations to physical activity. Physical fitness has been shown to elicit a more resilient immune defense and greater stress protection. In response to acute mental stress, physical fitness is associated with diminished pro-inflammatory cytokine responses (TNF-α and IL-6) (Hamer and Steptoe, 2007). This effect is not only seen in aerobic activity, but also with resistance exercise. In particular, the attenuated inflammatory response to training is a component of the repeated bout effect, which stipulates that when the same exercise (Nosaka et al., 2001) is performed in a subsequent resistance training bout, the muscle damage response is attenuated. Ultimately, IL-6 and IL-10 are important in recovery, and their blunted release following chronic training can be a demonstration of the repeated bout effect that signifies a reduction in the stress response to resistance training. Further investigation into the benefits of resistance exercise and stress is warranted, although initial reports are promising and parallel the benefits of aerobic exercise.

Finally, the interaction of obesity and psychological stress on the development of CVD is not fully understood. Future examination of the mechanisms contributing to endothelial dysfunction and the links between obesity, psychological stress and CVD will undoubtedly lead to the development of more specific and effective strategies, such as physical activity training, to address the ill effects of stress on CVD.

### Conflict of interest statement

The authors declare that the research was conducted in the absence of any commercial or financial relationships that could be construed as a potential conflict of interest.
